# Rethinking favorable outcomes: “return to baseline” as an additional outcome in ischemic stroke

**DOI:** 10.3389/fneur.2026.1866829

**Published:** 2026-10-19

**Authors:** Julia-Laura Schulz, Arno Reich, Charlotte S. Weyland, Hani Ridwan, Dimah Hasan, Rebecca May, Martin Wiesmann, Omid Nikoubashman, João Pinho

**Affiliations:** 1 Department of Diagnostic and Interventional Neuroradiology, University Hospital RWTH Aachen, Aachen, Germany; 2 Department of Neurology, University Hospital RWTH Aachen, Aachen, Germany; 3 Department of Neuroradiology, University Hospital Bielefeld, Bielefeld, Germany

**Keywords:** disability, functional outcomes, modified Rankin Scale, return to baseline, stroke, stroke recovery

## Abstract

**Background:**

Stroke outcomes are commonly assessed using the modified Rankin Scale (mRS), dichotomized into “favorable” (0–2) and “unfavorable” (3–6). Many elderly patients present with pre-stroke mRS scores of 3–5, making “favorable” outcomes unattainable and complicating treatment evaluation.

**Methods:**

This is a monocentric, retrospective analysis of ischemic stroke patients in our prospective registry (2010–2022). Outcomes include mRS scores at discharge and 90 days post-stroke. Descriptive and inferential statistics were applied with appropriate tests for categorical and ordinal variables. Multivariable logistic regression was used to model return to pre-stroke mRS and death by 90 days, adjusted for pre-stroke mRS group, age, sex, NIHSS on admission, treatment modality and vascular territory. Statistical significance was defined as *p* < 0.05.

**Results:**

Of 3,724 ischemic stroke patients admitted, 2,070 met inclusion criteria (median age 75.7). Among those with favorable pre-stroke mRS (0–2, *n* = 1,827), 47.6% achieved favorable outcomes at discharge and 47.5% at 90 days. By definition, none with unfavorable pre-stroke mRS achieved favorable outcomes. At discharge, return to baseline mRS occurred in 16.2% with favorable pre-stroke mRS, compared to 19.3% with unfavorable (*p* = 0.216). 90 days post-stroke, return to baseline occurred in 21.8% with favorable pre-stroke mRS, compared to 11.9% with unfavorable (*p* < 0.001). Redefining favorable outcome to include return to pre-stroke mRS increased rates from 42.0 to 44.3% at discharge and from 41.9 to 43.3% 90 days post-stroke (*p* < 0.001). In multivariable analysis a pre-stroke mRS of 3–5 was associated with death by 90 days (OR 4.40, 95% CI 3.18–6.10) but not with return to baseline in all patients (OR 1.02, 95% CI 0.65–1.58); among survivors it was associated with a higher probability of return to baseline (OR 2.34, 95% CI 1.40–3.91).

**Conclusion:**

The traditional dichotomization of mRS does not reflect the wide range of pre-stroke status. Incorporating “return to pre-stroke mRS score” as a favorable outcome metric alongside the conventional dichotomy provides a more individualized measure, improving treatment evaluation and supporting tailored care strategies, particularly for patients with higher pre-stroke mRS scores.

## 1 Introduction

Ischemic stroke is a leading cause of long-term disability and morbidity worldwide, accounting for approximately 62.4% of all stroke cases globally (1, 2). More than 90% of the global stroke burden is attributable to modifiable risk factors including hypertension, smoking, diabetes mellitus, poor diet and cardiac causes such as atrial fibrillation (1).

Patient outcomes are commonly assessed using the modified Rankin Scale (mRS), first introduced in the 1950s and subsequently refined for broader use (3, 4). The mRS is a standardized and well-recognized classification that evaluates the level of functional independence, degree of impairment in daily life activities and overall recovery (5). Dichotomizing the mRS score into “favorable” and “unfavorable” is one of the most widely used measures to evaluate the effectiveness of different stroke therapies. A “favorable” outcome is usually defined by an mRS score of 0–2, while an mRS score of 3–6 indicates an “unfavorable” outcome (6–8). While this dichotomization has practical value for trial design and sample-size calculation, it treats all “unfavorable” outcomes as clinically equivalent, collapsing a wide spectrum of functional states into a single category. This simplification can obscure treatment effects that hinder the restoration of full independence (6).

Elderly patients with comorbidities and pre-stroke disabilities make up a substantial proportion of ischemic stroke cases, and their pre-stroke mRS scores are often not ideal, sometimes reaching 3–5, indicating an already “unfavorable” status (1, 9). Hence, even with successful treatment, many of these patients may not achieve a “favorable” mRS outcome of 0–2 (10). A premorbid mRS cutoff has led to the exclusion of over a third of stroke patients older than 65 from most trials (11). This complicates the evaluation of treatment effectiveness.

This study sets out to demonstrate that even a formally “unfavorable” outcome score can still reflect successful treatment in patients with relevant pre-stroke disabilities or comorbidities and unfavorable pre-stroke mRS. We evaluate “return to pre-stroke mRS score” as an additional metric for assessing the effectiveness of stroke therapies.

## 2 Methods

This study is a monocentric, retrospective data analysis of ischemic stroke patients included in our local center-based prospective stroke registry, covering patients admitted from January 2010 to December 2022. Our analysis was approved by our local ethics committee (approval reference EK 335/15). The requirement for patient-signed consent for this analysis was waived by the committee due to its retrospective nature.

All patients receive regular care according to national and international recommendations, with treatment decisions made through interdisciplinary collaboration. Data is collected as part of the clinical routine. Key data points encompass patient demographics, clinical presentation measured by the NIHSS and mRS score, treatment details, and comorbidities. Baseline functional status is captured through the mRS score prior to the index stroke (“pre-stroke mRS”). Outcome data include the mRS score at discharge and at 90 days post-stroke, collected during in-person or telephone follow-ups. These assessments are performed by trained raters using a standardized structured interview format.

Consecutive patients with a diagnosis of acute ischemic stroke admitted between January 2010 and December 2022 were eligible. Patients were included only if the pre-stroke mRS, mRS on admission, mRS at discharge and 90 days post-stroke were all documented; patients missing any of these four scores were excluded. No restriction was applied on stroke severity, on the degree of premorbid disability or on the treatment received, so that the cohort reflects the full spectrum of patients treated at a stroke center. Patients presenting with a recurrent stroke were eligible, their pre-stroke mRS then refers to the functional state immediately before the index event; a previous ischemic stroke was recorded separately as a comorbidity.

For the purposes of this study, we assessed and compared the evaluation of the mRS score throughout the hospital stay and 90 days post-stroke. Patients therefore were divided into two groups based on their pre-stroke mRS: favorable (0–2) and unfavorable (3–5). We then evaluated how many patients in each group returned to their baseline pre-stroke mRS after their treatment. “Return to baseline” was defined as achieving the same mRS score as prior to the index stroke at discharge or 90 days post-stroke, respectively. Return to baseline is therefore a point-wise, directional measure, which registers recovery back to a patient’s own premorbid level and does not distinguish the extent of deterioration below it.

Acute treatment was classified into four categories: intravenous thrombolysis alone, intravenous thrombolysis combined with endovascular treatment, endovascular treatment alone, and no reperfusion therapy. Carotid revascularization via stenting, angioplasty or thrombendarteriectomy and decompressive surgery via hemicraniectomy or suboccipital decompression were recorded separately. For patients undergoing mechanical thrombectomy, procedural success was graded with the Thrombolysis in Cerebral Infarction (TICI) score, and successful reperfusion was defined as TICI 2b, 2c or 3. The affected vascular territory was classified as anterior circulation, posterior circulation, or multiple territories.

Three multivariable logistic regression models were fitted: return to pre-stroke mRS at 90 days post-stroke in all patients (Model 1), death at 90 days (Model 2) and return to pre-stroke mRS at 90 days post-stroke restricted to patients alive at 90 days post-stroke (Model 3). All three models were adjusted for pre-stroke mRS group (0–2 vs. 3–5), age, sex, NIHSS on admission, treatment modality and vascular territory. Vascular risk factors were not included as covariates in the main models. In a sensitivity analysis additionally adjusted for arterial hypertension, atrial fibrillation and diabetes mellitus, the odds ratios for pre-stroke mRS group changed by less than 0.1 in all three models, so the simpler specification is reported throughout. Because reperfusion grade is defined only for thrombectomy patients, TICI was not included into the three main models; instead, a separate model restricted to endovascular patients was fitted with successful reperfusion as an additional covariate. Patients with a missing NIHSS (*N* = 37) were excluded from the regression models by listwise deletion, consistent with the no-imputation approach; the endovascular model additionally excluded patients with missing reperfusion status.

Because treatment allocation in this cohort followed interdisciplinary clinical judgment that itself takes a patient’s premorbid functional status into account, treatment modality may act partly as a mediator rather than purely as a confounder of the association between pre-stroke mRS and outcome. We therefore report outcomes both treatment-stratified and treatment-adjusted, the latter reflecting the association in the multivariable analysis conditional on the treatment received. The two are intended as complementary perspectives rather than competing estimates of a single effect. The treatment coefficients themselves should accordingly be read as associations with the treatment actually received, not as independent causal effects of treatment.

### 2.1 Statistics

Parametric data are presented as mean ± standard deviation (SD), non-parametric as median with interquartile range (IQR). Data distribution was evaluated using the Shapiro–Wilk test. Chi-square tests were employed to compare categorical data, while McNemar tests were conducted to evaluate the difference in paired categorical data. Mann–Whitney U tests were used for ordinal data comparisons when normal distribution was not observed. Odds ratios (OR) are reported with 95% confidence intervals (CI). A *p*-value below the alpha level of 0.05 was considered statistically significant. All statistical analyses were performed with IBM SPSS Statistics, version 29 (IBM, Armonk, United States). This report adheres to the Strengthening the Reporting of Observational Studies in Epidemiology (STROBE) guidelines to ensure transparent reporting (12). No imputation was applied for missing data, ensuring the integrity of the dataset.

## 3 Results

A total of 3,724 ischemic stroke patients were admitted to our center over this 12-year period. For our study, only patients with known pre-stroke mRS score, known mRS score on admission, on discharge and 90 days after stroke were considered. After excluding 1,654 patients due to missing pre-stroke and/or 90-day mRS values, the final study population consisted of 2,070 patients.

Median age of our population was 75.7 years (IQR, 64.4–82.9) and 47.4% (982) were female. Common comorbidities included hypertension (80.5%), atrial fibrillation (38.8%) and diabetes mellitus (26.5%). Further details on baseline characteristics, comorbidities, and functional outcomes can be found in Table 1.

**Table 1:** Baseline characteristics, comorbidities, stroke severity, treatment, procedural time metrics, and functional outcomes by pre-stroke modified Rankin Scale groups (0–2 vs. 3–5, *N* = 2,070).

Variable	Pre-stroke mRS 0–2 [*N* = 1,827]	Pre-stroke mRS 3–5 [*N* = 243]	*p*-value
Age (years) [median]	74.0 (IQR 62.8–81.5)	84.3 (IQR 78.4–89.6)	< 0.001
Female sex [*n*]	832 (45.5%)	150 (61.7%)	< 0.001
Intravenous thrombolysis [*n*]	1,293 (70.8%)	156 (64.2%)	0.036
Neuroradiological intervention [*n*]	1,034 (56.6%)	152 (62.6%)	0.078
Successful recanalization^†^ [*n*]	923*^°^*/1,034 (90.3%)	136/152 (89.5%)	0.745
Arterial hypertension [*n*]	1,448^§^ (79.3%)	218^‡^ (90.1%)	< 0.001
Atrial fibrillation [*n*]	654^§^ (35.8%)	149^‡^ (61.6%)	< 0.001
Diabetes mellitus [*n*]	463^§^ (25.4%)	85^‡^ (35.1%)	0.001
Dyslipidemia [*n*]	566^§^ (31.0%)	72^‡^ (29.8%)	0.690
Adiposity [*n*]	433^§^ (23.7%)	52^‡^ (21.5%)	0.440
Nicotine abuse [*n*]	446^§^ (24.4%)	24^‡^ (9.9%)	< 0.001
Previous ischemic stroke [*n*]	317^§^ (17.4%)	86^‡^ (35.5%)	< 0.001
mRS score pre-stroke [median]	0 (IQR 0–0)	3 (IQR 3–4)	< 0.001
mRS score on admission [median]	4 (IQR 3–4)	4 (IQR 4–5)	< 0.001
mRS score on discharge [median]	3 (IQR 1–5)	6 (IQR 4–6)	< 0.001
mRS 90 days post-stroke [median]	3 (IQR 1–5)	6 (IQR 5–6)	< 0.001
mRS difference discharge–admission [median]	–1 (IQR –2 to 1)	1 (IQR 0–2)	< 0.001
mRS difference 90 days–admission [median]	–1 (IQR –2 to 1)	1 (IQR 1–2)	< 0.001
NIHSS on admission^#^ [median]	9 (IQR 4–17)	15 (IQR 9–20)	< 0.001
Anterior circulation [*n*]	1,452 (79.5%)	207 (85.2%)	
Posterior circulation [*n*]	251 (13.7%)	22 (9.1%)	
Multiple territories [*n*]	124 (6.8%)	14 (5.8%)	
Overall territory distribution			0.092
IVT alone [*n*]	756 (41.4%)	87 (35.8%)	
IVT + endovascular treatment [*n*]	537 (29.4%)	69 (28.4%)	
Endovascular treatment alone [*n*]	497 (27.2%)	83 (34.2%)	
No reperfusion therapy [*n*]	37 (2.0%)	4 (1.6%)	
Overall treatment distribution			0.135
Door-to-needle^*^ (minutes) [median]	30 (IQR 25.0–40.0) [*N* = 833]	29 (IQR 25.0–36.0) [*N* = 95]	0.451
CT-to-puncture^*^ (minutes) [median]	48.5 (IQR 35.0–62.0) [*N* = 1,030]	48.5 (IQR 36.8–61.2) [*N* = 150]	0.978
Puncture-to-recanalization^*^ (minutes) [median]	37 (IQR 27.0–57.0) [*N* = 991]	41.5 (IQR 27.0–62.2) [*N* = 146]	0.198
Door-to-recanalization^*^ (minutes) [median]	113 (IQR 95.0–148.0) [*N* = 938]	121.5 (IQR 96.8–150.5) [*N* = 138]	0.371
Carotid revascularization^#^ (stenting/angioplasty/TEA) [*n*]	285 (15.7%)	21 (8.7%)	0.004
Decompressive surgery [*n*]	70 (3.8%)	0 (0.0%)	< 0.001

Percentages are rounded to the first decimal place and based on valid cases. IQR, interquartile range; IVT, intravenous thrombolysis; mRS, modified Rankin Scale; NIHSS, National Institutes of Health Stroke Scale; TEA, thrombendarteriectomy; TICI, Thrombolysis in Cerebral Infarction. †Successful recanalization defined as TICI 2b/2c/3. ‡Missing information for 1 patient. §Missing information for 2 patients. °Missing information for 12 patients. #Missing information on NIHSS for 37 patients, carotid revascularization for 13. *Procedural time metrics are defined only for treated patients, with denominators given in brackets; door-to-needle time was frequently unrecorded when thrombolysis was started at a referring hospital.

Acute treatment consisted of intravenous thrombolysis alone in 843 patients (40.7%), intravenous thrombolysis combined with endovascular treatment in 606 (29.3%), endovascular treatment alone in 580 (28.0%) and no reperfusion therapy in 41 (2.0%). Carotid revascularization was performed in 306 patients (14.8%) and decompressive surgery in 70 (3.4%). Among the patients treated endovascularly, successful reperfusion (TICI 2b–3) was achieved in 1,059 (90.2%). The infarct involved the anterior circulation in 1,659 patients (80.1%), the posterior circulation in 273 (13.2%) and multiple territories in 138 (6.7%). The distribution of treatment modalities did not differ significantly between the two pre-stroke mRS groups (*p* = 0.135), nor did the overall distribution of vascular territories (*p* = 0.092), although anterior circulation strokes were numerically more frequent (85.2% vs. 79.5%) and posterior circulation strokes less frequent (9.1% vs. 13.7%) in the unfavorable pre-stroke mRS group (Table 1).

1,827 (88.3%) patients had a pre-stroke mRS score of 0–2 and 243 (11.7%) had a pre-stroke mRS score of 3–5. Upon admission, 374 (18.1%) patients had an mRS score of 0–2, while 1,696 (81.9%) had an mRS score of 3–5. At discharge, 869 (42.0%) patients had an mRS score of 0–2, while 1,201 (58.0%) had an mRS score of 3–6. 90 days after stroke, 867 (41.9%) patients had an mRS score of 0–2, while 1,203 (58.1%) had an mRS score of 3–6. Focusing exclusively on the favorable pre-stroke mRS group (mRS 0–2), 47.6% achieved a favorable outcome (mRS 0–2) at discharge, and 47.5% maintained a favorable outcome 90 days post-stroke. By definition, none of the individuals in the unfavorable pre-stroke mRS group achieved a favorable outcome.

At discharge, 16.2% of patients in the favorable pre-stroke mRS group (0–2) returned to their baseline mRS, compared to 19.3% in the unfavorable group (3–5) (*p* = 0.216). 90 days post-stroke, 21.8% of patients in the favorable pre-stroke mRS group (0–2) returned to their baseline mRS, compared to 11.9% in the unfavorable group (3–5) (*p* < 0.001).

Outcomes by treatment modality are shown in Table 2. Return to pre-stroke mRS at 90 days post-stroke was most frequent after intravenous thrombolysis alone (32.5% in the favorable vs. 17.2% in the unfavorable pre-stroke mRS group, *p* = 0.003) and least frequent after endovascular treatment alone (10.5% vs. 6.0%, *p* = 0.209). At discharge, the only significant difference between pre-stroke mRS groups was in patients treated with thrombolysis combined with endovascular treatment, where more patients with unfavorable pre-stroke mRS had returned to baseline (18.8% vs. 8.4%, *p* = 0.005). Mortality at 90 days was markedly higher in the unfavorable pre-stroke mRS group after thrombolysis alone (59.8% vs. 11.2%), thrombolysis combined with endovascular treatment (65.2% vs. 28.7%) and endovascular treatment alone (79.5% vs. 35.0%, all *p* < 0.001). The same direction was seen after no reperfusion therapy (2 of 4 vs. 13 of 37, *p* = 0.615).

**Table 2:** Return to pre-stroke mRS and 90-day mortality by treatment modality and pre-stroke mRS (0–2 vs. 3–5, *N* = 2,070).

Treatment	Pre-stroke mRS	Return to pre-stroke mRS at discharge	*p*-value	Return to pre-stroke mRS at 90 days	*p*-value	Death at 90 days	*p*-value
IVT alone	0–2 [*N* = 756]	29.1%	0.943	32.5%	0.003	11.2%	< 0.001
	3–5 [*N* = 87]	28.7%		17.2%		59.8%	
EVT + IVT	0–2 [*N* = 537]	8.4%	0.005	17.3%	0.230	28.7%	< 0.001
	3–5 [*N* = 69]	18.8%		11.6%		65.2%	
EVT alone	0–2 [*N* = 497]	5.6%	0.162	10.5%	0.209	35.0%	< 0.001
	3–5 [*N* = 83]	9.6%		6.0%		79.5%	
No reperfusion therapy	0–2 [*N* = 37]	8.1%	0.348	18.9%	1.000	35.1%	0.615
	3–5 [*N* = 4]	25.0%		25.0%		50.0%	

Percentages are rounded to the first decimal. EVT, endovascular treatment; IVT, intravenous thrombolysis; mRS, modified Rankin Scale.

Figure 1 shows the proportion of 90-day survivors by pre-stroke mRS who returned to their pre-stroke mRS, at discharge and at 90 days. At discharge, 20.8% of patients in the favorable pre-stroke mRS group (0–2) returned to their baseline mRS, compared to 50.0% in the unfavorable group (3–5) (*p* < 0.001). 90 days post-stroke, 28.4% of patients in the favorable pre-stroke mRS group (0–2) returned to their baseline mRS, compared to 37.2% in the unfavorable group (3–5) (*p* = 0.096). The two groups therefore converged over time, moving in opposite directions, so the initial advantage of the unfavorable group narrowed and lost significance by 90 days.

**Figure 1:**
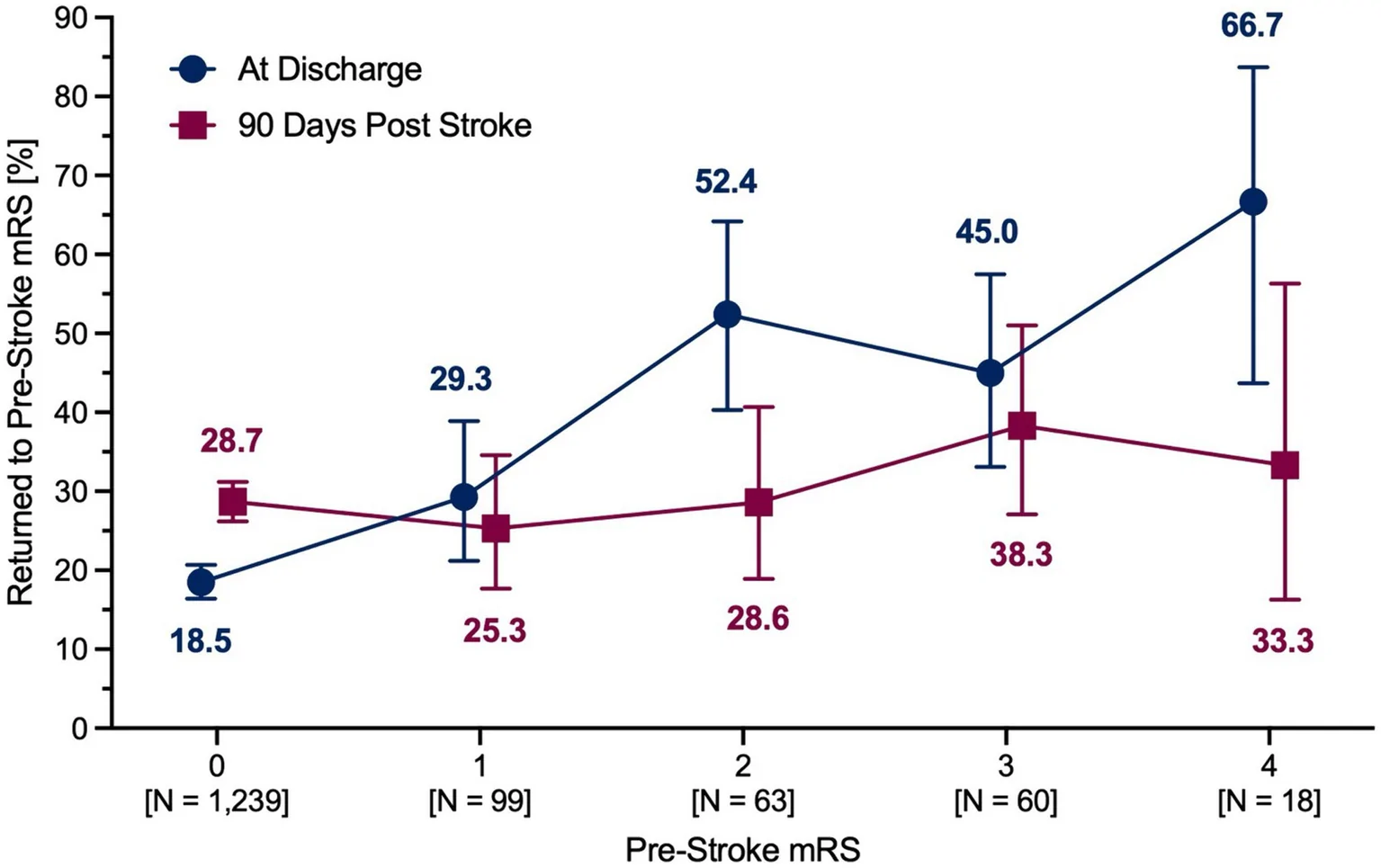
Return to pre-stroke mRS distribution at discharge and 90 days by pre-stroke mRS score, only considering patients who survived the 90-day mark (*N* = 1,479). Percentages are rounded to the first decimal place. Bars are 95% Wilson confidence intervals. Denominators are patients alive at 90 days. mRS, modified Rankin Scale.

Adding patients with an unfavorable pre-stroke condition who returned to their pre-stroke mRS to the pool of “favorable” outcomes yields the following results (Figure 2): at discharge, the favorable outcome rate significantly increased from 42.0 to 44.3% (*p* < 0.001). At 90 days, favorable outcome rate significantly increased from 41.9 to 43.3% (*p* < 0.001).

**Figure 2:**
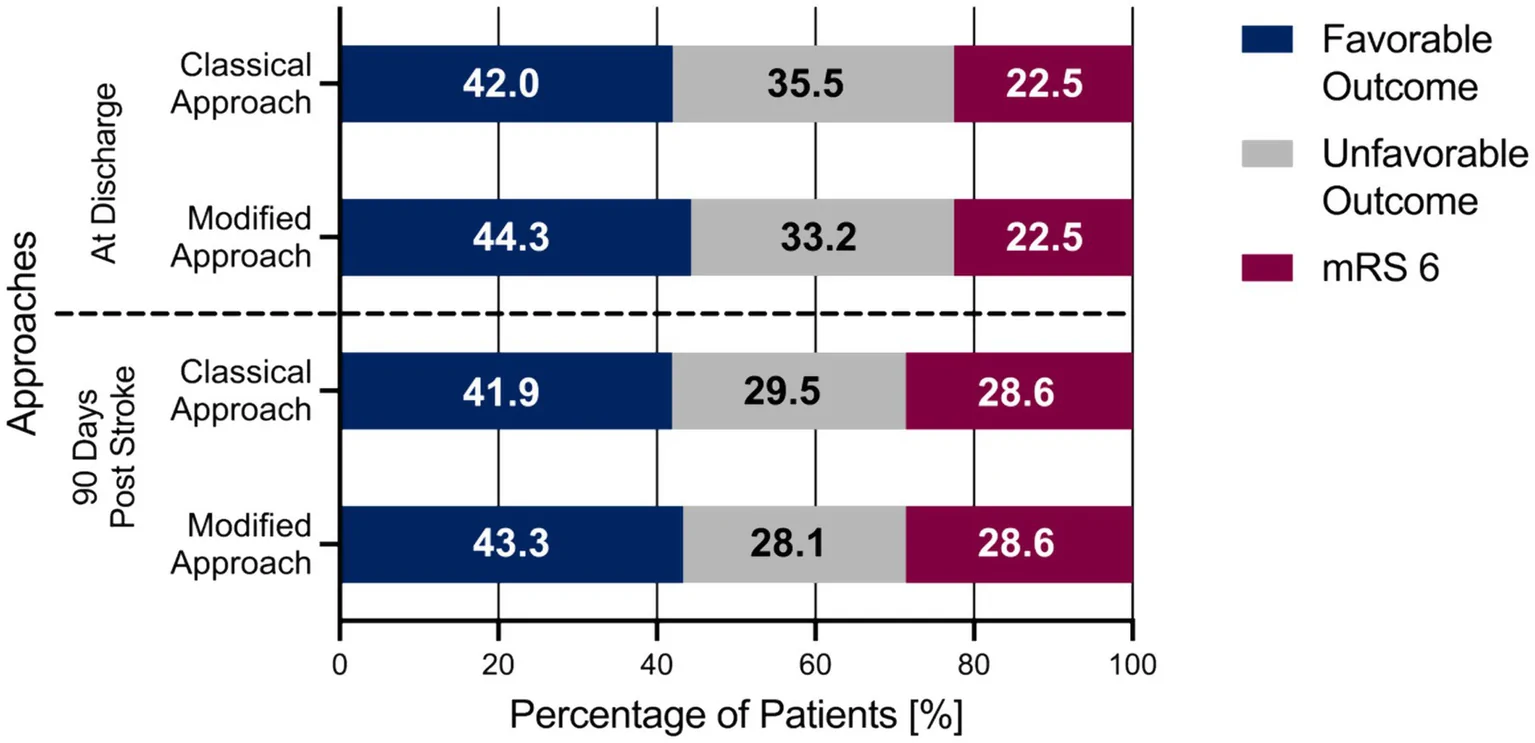
Comparison of outcome distributions by approach: classical (favorable mRS 0–2) vs. modified (favorable mRS 0–2 or return to pre-stroke mRS) at discharge and 90 days post-stroke (*N* = 2,070). Percentages are rounded to the first decimal place. mRS, modified Rankin Scale.

The distribution of mRS scores across all four timepoints and the transition from pre-stroke mRS to mRS at 90 days post-stroke are shown in Figure 3. The distribution of mRS scores shifted from 88.3% of patients with a pre-stroke mRS of 0–2 to 18.1% on admission, recovering to 42.0% at discharge and 41.9% at 90 days post-stroke, while the proportion of deceased patients rose from 22.5 to 28.6% (Figure 3a). Return to pre-stroke mRS at 90 days post-stroke declined steadily with increasing premorbid disability, from 22.4% in patients with a pre-stroke mRS score of 0 to 19.4% at 1, 16.1% at 2, 13.2% at 3 and 9.0% at 4, while 90-days mortality rose across the same range from 21.9 to 73.1% (Figure 3b). The two patients with a pre-stroke mRS of 5 were omitted from the figure; both died.

**Figure 3:**
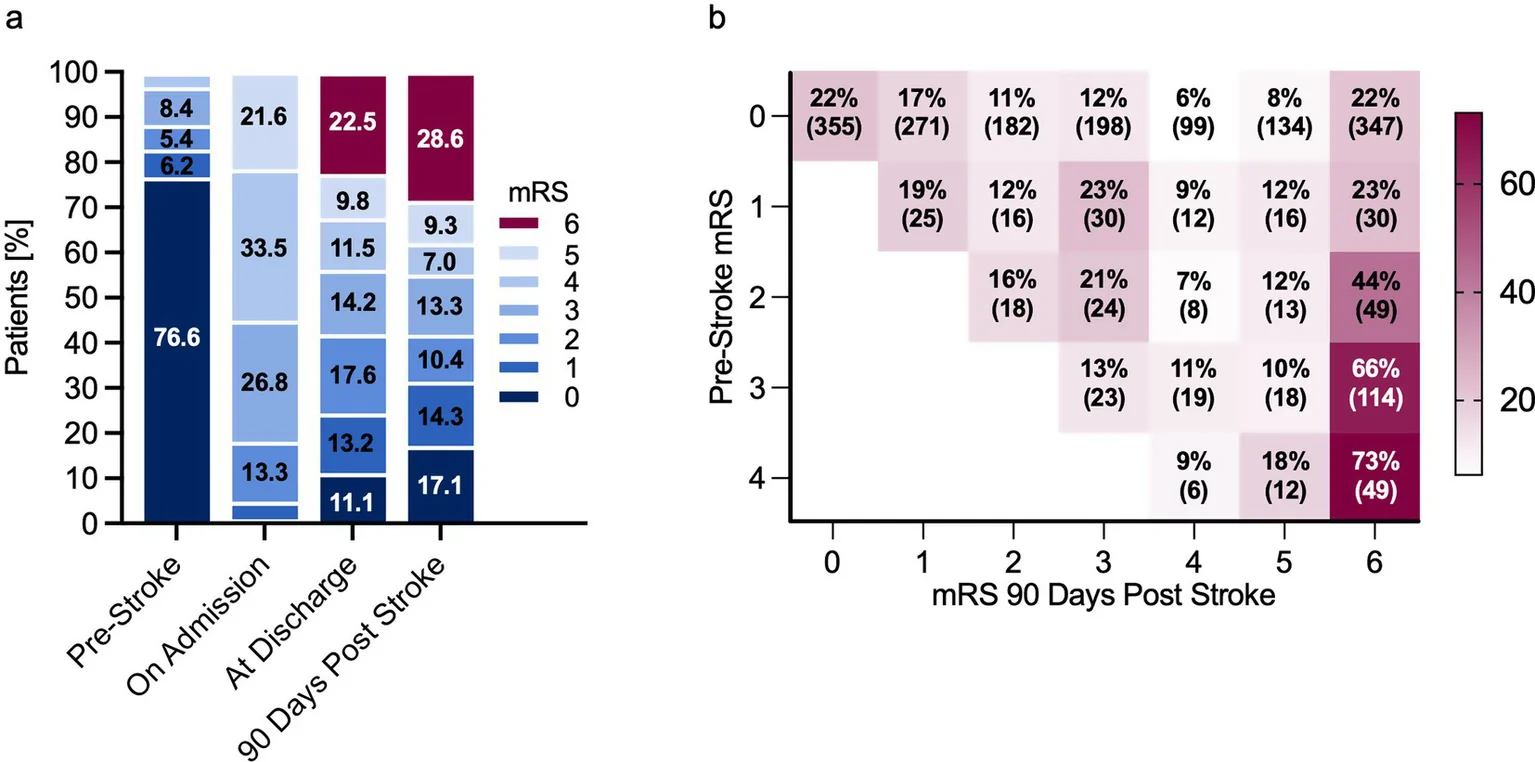
Distribution of mRS scores across four timepoints **(a)** and transition from pre-stroke to 90 days post stroke **(b)**. **(a)** mRS proportion of all patients (*N* = 2,070) before stroke, on admission, at discharge and 90 days post stroke. Columns sum to 100% subject to rounding. **(b)** mRS transition from pre-stroke to 90 days post stroke, shown as row percentage (*n*). Cells are shaded by row percentage. Two patients with pre-stroke mRS 5 are omitted (*N* = 2,068); both died. Empty cells indicate no patient made that transition. mRS: modified Rankin Scale.

In multivariable analysis (Table 3), an unfavorable pre-stroke mRS was not associated with return to baseline at 90 days post-stroke in the cohort (OR 1.02, 95% CI 0.65–1.58, *p* = 0.943), but was strongly associated with death at 90 days post-stroke (OR 4.40, 95% CI 3.18–6.10, *p* < 0.001). Among patients alive at 90 days post-stroke, an unfavorable pre-stroke mRS was associated with higher odds of returning to baseline (OR 2.34, 95% CI 1.40–3.91, *p* = 0.001). Higher NIHSS on admission and endovascular treatment alone were independently associated with both a lower chance of returning to baseline and higher mortality, as was involvement of multiple territories.

**Table 3:** Multivariable logistic regression for return to pre-stroke mRS and 90-day mortality.

Variable	Model 1 return to baseline at 90 days, all patients^#^ OR (95% CI) [*N* = 2,033]	*p*-value	Model 2 death at 90 days^#^ OR (95% CI) [*N* = 2,033]	*p*-value	Model 3 return to baseline at 90 days, only survivors^†^ OR (95% CI) [*N* = 1,462]	*p*-value
Pre-stroke mRS 3–5	1.02 (0.65–1.58)	0.943	4.40 (3.18–6.10)	< 0.001	2.34 (1.40–3.91)	0.001
Age, per year	0.98 (0.97–0.99)	< 0.001	1.03 (1.02–1.04)	< 0.001	0.99 (0.98–1.00)	0.072
Female Sex	0.87 (0.69–1.10)	0.237	0.92 (0.73–1.15)	0.447	0.83 (0.65–1.06)	0.132
NIHSS, per point	0.90 (0.88–0.92)	< 0.001	1.10 (1.08–1.11)	< 0.001	0.93 (0.91–0.95)	< 0.001
IVT + EVT	0.98 (0.72–1.34)	0.905	1.31 (0.96–1.78)	0.094	1.02 (0.73–1.41)	0.926
EVT alone	0.45 (0.32–0.63)	< 0.001	2.01 (1.49–2.72)	< 0.001	0.52 (0.37–0.74)	< 0.001
No reperfusion therapy	0.90 (0.39–2.08)	0.809	1.87 (0.86–4.09)	0.117	0.98 (0.41–2.35)	0.963
Posterior circulation	0.83 (0.59–1.15)	0.260	1.18 (0.82–1.69)	0.375	0.90 (0.64–1.27)	0.554
Multiple territories	0.49 (0.29–0.83)	0.008	1.62 (1.04–2.54)	0.034	0.53 (0.31–0.92)	0.024

Odds ratios are adjusted for all variables listed. Reference categories: pre-stroke mRS 0–2, male sex, intravenous thrombolysis alone, anterior circulation. CI, confidence interval; EVT, endovascular treatment; IVT, intravenous thrombolysis; mRS, modified Rankin Scale; NIHSS, National Institutes of Health Stroke Scale; OR, odds ratio. # Excluded 37 patients for missing information on NIHSS. † Restricted to patients alive at 90 days (*N* = 1,479), excluded 17 patients for missing information on NIHSS.

Within the additional model restricted to endovascularly treated patients (N = 1,154, after excluding 32 patients for missing information on NIHSS or reperfusion status) and further adjusted for reperfusion grade, successful reperfusion (TICI 2b/2c/3) was associated with return to pre-stroke mRS at 90 days post-stroke (OR 3.46, 95% CI 1.37–8.72, *p* = 0.009). Among the 735 patients alive at 90 days post-stroke the point estimate was similar but no longer significant (OR 2.49, 95% CI 0.96–6.47, *p* = 0.062). In this subgroup an unfavorable pre-stroke mRS was again associated with higher odds of returning to baseline among survivors (OR 2.36, 95% CI 1.14–4.88, *p* = 0.020).

## 4 Discussion

This study demonstrates that an mRS score of 3–5 that formally signifies an “unfavorable” outcome can still reflect successful treatment, particularly in patients with pre-stroke disabilities. Considering “return to baseline” as an additional metric provides a more nuanced perspective on treatment efficacy, highlighting the importance of individualized recovery baselines. This applies to 243 of 2,070 patients in our cohort (11.7%), for whom the conventional endpoint was determined before the stroke occurred and no treatment effect could have moved them into the favorable category.

A key finding of our study is that among the patients with a favorable pre-stroke condition (mRS 0–2), 48% achieved a favorable outcome (mRS 0–2) at 90 days post-stroke, but only 22% returned to their pre-stroke condition. In patients with an unfavorable pre-stroke condition (mRS 3–5), a significantly lower share of 12% returned to their pre-stroke condition. At first glance, this appears to reflect a lower capacity of already disabled patients to recover after ischemic stroke. However, every 8th patient in the unfavorable pre-stroke mRS group manages to come back to pre-stroke level. The decline from 19% at discharge to 12% at 90 days in the unfavorable group was primarily driven by mortality after discharge rather than functional deterioration. Return to baseline rates at discharge did not differ significantly between groups (*p* = 0.216), while mortality in the unfavorable group increased from 50% at discharge to 68% at 90 days post-stroke, compared with 19 to 23% in the favorable group. The crude comparison at 90 days therefore reflects competing risk rather than a recovery deficit, as mortality removes patients from the denominator before recovery can be observed. When only considering patients who survived the 90-days-mark, return to baseline rates were in fact higher in the unfavorable group at discharge (50% vs. 21%, *p* < 0.001) and no longer differed significantly at 90 days (37% vs. 28%, *p* = 0.096; Figure 1). Among patients who survive, therefore, a higher pre-stroke mRS score does not predict less recovery relative to their own baseline; what it predicts is death.

Two mechanisms plausibly explain why survivors with premorbid disability returned to baseline more often, and neither is a recovery advantage. Return to baseline is a relative endpoint, and the absolute functional gain it requires decreases as premorbid disability increases. For example, a patient whose baseline is mRS 4 must recover to mRS 4, whereas a patient whose baseline is 0 must be left with no residual deficit. In addition, given that 67.9% of patients with a pre-stroke mRS of 3–5 died within 90 days, those who survived represent a selected subset of patients admitted with premorbid disability. What our data establish, therefore, is the negative claim, which is the one relevant to outcome reporting. Once mortality is accounted for, no recovery penalty attributable to premorbid disability is detectable. This is also why return to baseline rates within treatment modalities show no such advantage (Table 2). Those rates retain non-survivors in the denominator and therefore combine recovery with mortality; accordingly, the two significant differences between pre-stroke mRS groups run in opposite directions, while mortality was consistently higher in the unfavorable group across all three treated strata (*p* < 0.001 for each). The gradient across the full range of premorbid function makes the same point from the other direction. Return to baseline fell modestly from 22.4% at a pre-stroke mRS of 0 to 9.0% at 4, while 90-day mortality more than tripled from 21.9 to 73.1% (Figure 3b).

These observations were confirmed in the multivariable analysis (Table 3). An unfavorable pre-stroke mRS was not independently associated with return to baseline at 90 days post-stroke (OR 1.02, 95% CI 0.65–1.58), but was strongly associated with death (OR 4.40, 95% CI 3.18–6.10), and among survivors with a higher chance of returning to baseline (OR 2.34, 95% CI 1.40–3.91). Premorbid disability therefore acts primarily as a risk factor for death rather than as an obstacle to functional recovery in those who survive. A proposed outcome measure must also respond to treatment, and return to baseline does. In the model restricted to endovascularly treated patients, successful reperfusion was associated with return to baseline at 90 days post-stroke (OR 3.46, 95% CI 1.37–8.72), with a similar point estimate when only considering patients who survived the 90-day-mark (OR 2.49, 95% CI 0.96–6.47, *p* = 0.062). The metric therefore tracks procedural success in the same direction as established endpoints, while remaining definable in patients for whom the conventional dichotomy is not. Our findings align with the MR CLEAN Registry, which reported a 27% rate of returning to baseline among pre-stroke dependent patients, defined as those with an mRS score of 3–5 before the onset of the current stroke, who underwent endovascular thrombectomy (13). Additionally, a pooled meta-analysis on thrombectomy in patients with pre-existing disability (mRS 2–5) reported a similar 30% return to baseline rate (14). Our crude rate of 11.9% is lower because our cohort is unselected for treatment and includes patients who received no reperfusion therapy, and because we required exact equality with the pre-stroke score; among survivors, our rate of 37.2% is closely comparable to both.

The HERMES collaboration adopted mRS shift analysis to address the limitations of dichotomized outcome measures, which had previously restricted the sensitivity of outcome assessments (11). However, even this approach does not account for a patient’s pre-stroke functional status, and thus cannot reliably determine return to baseline. This underscores the need for more individualized outcome metrics that reflect meaningful recovery on a patient-specific level. The concept of evaluating treatment success based on the return to a patient’s pre-stroke mRS score introduces an individualized framework for outcome assessment, addressing the inherent limitations of the traditional dichotomous approach (15). Incorporating this additional measure allows researchers and clinicians to assess treatment success more accurately without additional effort, especially in patients with relevant pre-stroke disabilities (14, 16, 17). The “return to baseline” metric emphasizes the importance of functional recovery relative to an individual’s baseline condition. When adding patients who returned to their pre-stroke mRS to the “favorable outcome” pool, favorable outcome rates increased from 42.0 to 44.3% at discharge and from 41.9 to 43.3% at 90 days post-stroke (*p* < 0.001). The increase is modest in the cohort as a whole, but it is concentrated entirely in the 11.7% of patients for whom the conventional endpoint was unattainable from the outset and who would otherwise be counted as treatment failures by definition (Figure 2). This pooled rate combines two distinct concepts: crossing the conventional threshold of functional independence, and preservation of a patient’s own prior level of function. The established dichotomy is defined by that threshold alone, so including within-group improvement makes the combined rate less precise in what it denotes. We therefore report return to baseline separately throughout, and regard the combined definition as a supplement to the conventional dichotomy rather than a replacement for it.

### 4.1 Limitations

While our study provides valuable insights into outcome measure definitions for acute ischemic stroke patients, several limitations should be considered. The retrospective design and single-center focus may limit generalizability of our results. It is important to consider that the mRS score at 90 days post-stroke in our hospital is typically collected via telephone follow-ups, which can introduce certain biases such as reporting and recall bias, potentially leading to underreporting or misreporting of patient recovery. Selection bias may have been introduced by the exclusion of a group with missing mRS values. Stroke etiology according to the TOAST classification and infarct size were not included as covariates in the present analysis; residual confounding from these established prognostic factors can therefore not be excluded. Treatment modality was entered as a covariate to separate premorbid function from measured treatment differences. However, treatment was not randomly allocated, and its distribution differed numerically by pre-stroke mRS group. If treatment decisions are themselves informed by premorbid disability, treatment modality may partly mediate rather than purely confound this relationship, and adjusting for it could then understate the total real-world association.

Moreover, using the mRS scale has its own limitations as it focuses mostly on the motor deficits that the patient incurs with minimal importance given to the cognitive disabilities. Therefore, patients scoring low on mRS might still suffer severe cognitive deficits and hence unfavorable outcomes that remain undetected (18). A further limitation of the scale is that it is ordinal rather than interval, so the functional distance between adjacent categories is not constant and a one-point change does not carry the same meaning across the scale. Return to baseline is less affected by this than a fixed threshold, since each patient is compared with their own previous state, but it does not eliminate the problem; weighting transitions by their functional importance would be a logical next step, although published utility weights value absolute health states rather than restoration of a patient’s own prior level, and our registry contains no quality-of-life or preference measures from which weights for this concept could be derived. This also means that equal point-wise recoveries need not represent equal functional change. For example, a patient returning from mRS 4 to a pre-stroke score of 3 and a patient returning from 5 to a pre-stroke score of 4 both count as return to baseline, although the two transitions may differ in functional and clinical significance. Whether recovery from one level is more likely to occur spontaneously without intervention than from another cannot be addressed in our cohort, as almost all patients received reperfusion therapy. As return to baseline is a relative endpoint, the functional gain it requires is smaller at higher pre-stroke mRS scores, so it should complement rather than replace absolute outcome measures.

However, the strengths of our study, including a large sample size and robust statistical methods, enhance the reliability of our findings. Future studies with multi-center designs could prove beneficial in further validating our findings and exploring the long-term effects of different treatments in patients across varying levels of pre-stroke disability.

## 5 Conclusion

This study shows that the traditional way of dichotomizing the mRS into favorable and unfavorable outcomes does not do justice to the wide range of pre-stroke status in daily clinical practice. The results support that an unfavorable mRS score does not necessarily imply treatment failure and can even represent a better outcome than a favorable mRS score.

For future research and clinical practice purposes, we recommend incorporating “return to baseline” alongside the conventional dichotomy as a favorable outcome metric. Aligning with the realities of diverse patient populations, it provides a more individualized measure. Implementing this modified approach could lead to improved treatment evaluation and efficacy, hence guide more tailored care strategies, particularly for patients with higher pre-stroke mRS scores. Moreover, as cognitive deficits are not adequately captured by the mRS, future outcome assessments should also consider complementary tools to reflect the full spectrum of stroke-related impairments. Because premorbid disability in our cohort predicted death far more strongly than it predicted failure to recover, reporting mortality and functional recovery separately is essential when outcomes are compared across groups with differing pre-stroke function.

## Data Availability

The raw data supporting the conclusions of this article will be made available by the authors, without undue reservation.

## References

[1] Feigin VL Norrving B Mensah GA. Global burden of stroke. Circ Res. (2017) 120:439–48. doi: 10.1161/CIRCRESAHA.116.308413, 28154096

[2] Collaborators GBDS. Global, regional, and national burden of stroke and its risk factors, 1990-2019: a systematic analysis for the global burden of disease study 2019. Lancet Neurol. (2021) 20:795–820. doi: 10.1016/S1474-4422(21)00252-0, 34487721 PMC8443449

[3] Rankin J. Cerebral vascular accidents in patients over the age of 60. I. General considerations. Scott Med J. (1957) 2:127–36. doi: 10.1177/003693305700200401, 13432825

[4] Lees KR Bath PM Schellinger PD Kerr DM Fulton R Hacke W . Contemporary outcome measures in acute stroke research: choice of primary outcome measure. Stroke. (2012) 43:1163–70. doi: 10.1161/STROKEAHA.111.641423, 22426313

[5] Banks JL Marotta CA. Outcomes validity and reliability of the modified Rankin scale: implications for stroke clinical trials: a literature review and synthesis. Stroke. (2007) 38:1091–6. doi: 10.1161/01.STR.0000258355.23810.c6, 17272767

[6] Ganesh A Luengo-Fernandez R Wharton RM Rothwell PM, Oxford Vascular Study. Ordinal vs dichotomous analyses of modified Rankin scale, 5-year outcome, and cost of stroke. Neurology. (2018) 91:e1951–60. doi: 10.1212/WNL.0000000000006554, 30341155 PMC6260198

[7] Jovin TG Chamorro A Cobo E de Miquel MA Molina CA Rovira A . Thrombectomy within 8 hours after symptom onset in ischemic stroke. N Engl J Med. (2015) 372:2296–306. doi: 10.1056/NEJMoa1503780, 25882510

[8] Saver JL Goyal M Bonafe A Diener HC Levy EI Pereira VM . Stent-retriever thrombectomy after intravenous t-PA vs. t-PA alone in stroke. N Engl J Med. (2015) 372:2285–95. doi: 10.1056/NEJMoa1415061, 25882376

[9] Gresham GE Phillips TF Wolf PA McNamara PM Kannel WB Dawber TR. Epidemiologic profile of long-term stroke disability: the Framingham study. Arch Phys Med Rehabil. (1979) 60:487–91. 508073

[10] Blum F Hager C Taufik H Wiesmann M Hasan D Reich A . Seeing the good in the bad: actual clinical outcome of thrombectomy stroke patients with formally unfavorable outcome. Neuroradiology. (2022) 64:1429–36. doi: 10.1007/s00234-022-02920-1, 35257206 PMC9177466

[11] Goyal M Menon BK van Zwam WH Dippel DW Mitchell PJ Demchuk AM . Endovascular thrombectomy after large-vessel ischaemic stroke: a meta-analysis of individual patient data from five randomised trials. Lancet. (2016) 387:1723–31. doi: 10.1016/S0140-6736(16)00163-X, 26898852

[12] Von Elm E Altman DG Egger M Pocock SJ Gotzsche PC Vandenbroucke JP . The strengthening the reporting of observational studies in epidemiology (STROBE) statement: guidelines for reporting observational studies. PLoS Med. (2007) 4:e296. doi: 10.1371/journal.pmed.0040296, 17941714 PMC2020495

[13] Goldhoorn RB Verhagen M Dippel DWJ Van der Lugt A Lingsma HF Roos Y . Safety and outcome of endovascular treatment in prestroke-dependent patients. Stroke. (2018) 49:2406–14. doi: 10.1161/STROKEAHA.118.022352, 30355090

[14] Bala F Beland B Mistry E Almekhlafi MA Goyal M Ganesh A. Endovascular treatment of acute ischemic stroke in patients with pre-morbid disability: a meta-analysis. J Neurointerv Surg. (2023) 15:343–9. doi: 10.1136/neurintsurg-2021-018573, 35292569

[15] Broderick JP Adeoye O Elm J. Evolution of the modified Rankin scale and its use in future stroke trials. Stroke. (2017) 48:2007–12. doi: 10.1161/STROKEAHA.117.017866, 28626052 PMC5552200

[16] Tanaka K Yamagami H Yoshimoto T Uchida K Morimoto T Toyoda K . Endovascular therapy for acute ischemic stroke in patients with Prestroke disability. J Am Heart Assoc. (2021) 10:e020783. doi: 10.1161/JAHA.121.020783, 34284599 PMC8475666

[17] Yang JC Bao QJ Guo Y Chen SJ Zhang JT Zhang Q . Endovascular thrombectomy in acute ischemic stroke patients with prestroke disability (mRS >/=2): a systematic review and meta-analysis. Front Neurol. (2022) 13:971399. doi: 10.3389/fneur.2022.971399, 36188370 PMC9520304

[18] Nair S Hurly J Saylor D. Evaluating the strengths and limitations of structured modified Rankin scale validation studies – a systematic review. J Stroke Cerebrovasc Dis. (2025) 34:108242. doi: 10.1016/j.jstrokecerebrovasdis.2025.108242, 39922249

